# Longitudinal study of the impact of three major regulations on the Korean pharmaceutical industry in the last 30 years

**DOI:** 10.1186/s12961-021-00797-9

**Published:** 2022-01-06

**Authors:** Seung In Um, Uy Dong Sohn, Sun-Young Jung, Seung-Hun You, Changone Kim, Sora Lee, Heesang Lee

**Affiliations:** 1 Korea Pharmaceutical and Bio-Pharma Manufacturers Association, Seoul, South Korea; 2 Department of Pharmacology, Chung-Ang University, Seoul, South Korea; 3 College of Pharmacy, Chung-Ang University, Seoul, South Korea; 4 Department of Global Innovative Drugs, Chung-Ang University, Seoul, South Korea; 5 Graduate School of Global Entrepreneurship, Keimyung University, Daegu, South Korea; 6 Graduate School of Management of Technology, Sungkyunkwan University, Suwon, South Korea

**Keywords:** Pharmaceutical industry, Innovation, Regulations, Patent, GMP, Drug pricing system

## Abstract

**Background:**

The pharmaceutical industry is heavily regulated. Partly for this reason, new drugs generally take over 10 years from the product development stage to market entry. Although regulations affect the pharmaceutical industry over a long period, previous studies investigating the impact of new regulatory policies have usually focused on the short period before and after implementing that policy. Therefore, the purpose of this study is to examine whether and how significantly regulatory policies affect long-term innovation in the pharmaceutical industry in Korea.

**Methods:**

This study focused on three significant regulatory policies: the introduction of the product patent system, changes in the Good Manufacturing Practice (GMP) system, and the Drug Expenditure Rationalization Plan (DERP). The study used interrupted time series (ITS) analysis to investigate the long-term impacts of the policies before and after implementation.

**Results:**

Our results show that introducing the product patent system in 1987 significantly increased the number of Korean patent applications. The effect of the revised GMP policies was also statistically significant, both before and after implementation and between pre-emptive companies and non-pre-emptive ones. However, due to the companies' negotiations with the regulatory authorities or the regulatory system that links drug approval and price evaluation, the DERP did not significantly delay new drug registration in Korea.

**Conclusion:**

This study showed that the policies of the product patent system, GMP policies, and DERP regulations have significantly encouraged pharmaceutical companies to strive to meet regulatory requirements and promote innovation in Korea. The study suggests that it is necessary for companies to pre-emptively respond to systemic changes in development and production strategies to deal with regulatory changes and achieve sustainable growth. Also, our study results indicate that since government policies motivate the innovative system of the pharmaceutical industry, governmental authorities, when formulating pharmaceutical policies, need to consider the impact on the long-term innovation of the industry.

## Background

The pharmaceutical industry develops new drugs to address unmet medical needs and extend lifespan [1]. Simultaneously, it contributes significantly to a country’s economy and promotes GDP growth due to knowledge-based technological innovation [2]. Thus, the pharmaceutical industry is a more high-technology, high-growth, and knowledge-based sector than most other industrial sectors. However, new drug development generally takes over 10–15 years, and each new drug has a low probability of success [3, 4]. Recently, the average overall cost of developing a new drug was estimated at US$ 2.8 billion [5]. One reason for the high research and development (R&D) costs is the tight and inflexible nature of the pharmaceutical regulations [6]. For example, Food and Drug Administration (FDA) regulations have caused smaller pharmaceutical firms in the United States to suffer reductions in research productivity [7]. In addition, clinical trials, which account for the most significant proportion of total R&D time due to strong safety and effectiveness regulations, have recently become more complex and costly [3].

Regulations are necessary to ensure pharmaceutical safety and effectiveness and the accuracy of the information given to customers. They are linked to the market responsibilities of manufacturers [8]. Pharmaceutical regulations have different goals that depend on the income level of the applicable country. Generally, low-income countries value the quality of medicines, middle-income countries value fiscal and industrial development, and high-income countries value innovation in new drug R&D [9]. Although many pharmaceutical regulations exist, some of the most critical regulatory policies that impact pharmaceutical innovation concern the patent system, Good Manufacturing Practice (GMP), and price controls [10–15]. A new innovative drug, the final output of innovation by the pharmaceutical industry, must be approved by the regulatory authorities at every development stage. If a drug that had not been validated for safety or efficacy were to be released without approval by the regulatory authority, or if a marketed drug was not controlled because of ineffective regulations, it would almost certainly be a disaster [16, 17].

The pharmaceutical industry, which is based on regulation, grows through innovation. Therefore, it is necessary to analyse any changes in a country’s regulatory policies and the long-term impact of such policy changes on innovation and growth in the pharmaceutical industry. This study investigates the long-term effects of three significant changes in pharmaceutical regulations over the 30 years from 1988 to 2017 in Korea, where the pharmaceutical industry expanded by about eightfold. The first significant change was the introduction of the product patent system in 1987; the second one was the changes to the GMP system in 1994, 2008, and 2014; and the final one was the Drug Expenditure Rationalization Plan (DERP), the new pharmacoeconomic evaluation system in 2006. We used the following three research questions to analyse the impacts of these three regulatory changes:

- Research question 1: Did the introduction of the product patent system increase the number of patent applications filed by the pharmaceutical industry in Korea?
- Research question 2: Did companies that pre-emptively invested in GMP facilities before mandatory GMP maintain sustainable growth in Korea?
- Research question 3: Did the DERP delay the introduction of new drugs in Korea?

### Product patent regulation and pharmaceutical innovation

The role of patents is to encourage innovation in biopharmaceuticals and accelerate the development of new drugs. The introduction and strengthening of the product patent system has shifted the focus of the pharmaceutical industry from imitation to innovation [18]. Prior research shows a positive correlation between product patents, new drugs, and R&D [10, 19, 20]. The patent system in the United States provides an incentive for R&D by protecting the exclusive right to recover profit for a considerable period [21]. The Indian pharmaceutical industry grew tenfold between introducing the patent laws in 1970 and the early 2000s [21].

Korea first enacted the Patent Law in 1946, then joined the Paris Convention in 1980 and the Patent Cooperation Treaty (PCT) in 1982. The Korean government revised the Patent Law to reflect the product patent system on 29 December 1986. Subsequently, the technological innovation of the domestic pharmaceutical industry was promoted by the revised laws in 1990 and 2001 [22]. Several studies have shown that since the 1980s, Korean patent applications have continued to increase [23, 24], and since 1992, the number of Korean patent applications has surpassed that of foreign applications [24].

Though many studies have analysed the impact of changes in the Korean patent system, most such studies investigated the effects of introducing a new patent system on the pharmaceutical industry by focusing on the short period before and after the introduction of the new system. For example, by conducting trend analysis of descriptive statistics, Lee and Yoon [23, 24] revealed an increase in the number of pharmaceutical patent applications.

As the first research question, we ask whether there were any changes in the number of Korean patent applications filed by the pharmaceutical industry over the 18 years from 1981 to 1998, which encompasses the introduction of the product patent system in Korea in 1987. An increase in patent applications under the new product patent system would be a cornerstone for long-term innovation in the Korean pharmaceutical industry.

### GMP regulations and the sustainable growth of pharmaceutical companies

GMP refers to regulations, codes, and guidelines for manufacturing final pharmaceutical products, raw materials, medical devices, and diagnostic products. Pharmaceutical companies worldwide must apply GMP to all manufacturing and quality control processes [17]. GMP regulation has resulted in smaller companies giving up on new drug innovation and instead focusing on me-too drug development. However, large companies create new drugs by steadily investing in R&D and manufacture the drugs following GMP regulations.

In Korea, as in the United States, Europe, and Japan, the GMP system has been continuously strengthened and revised through international harmonization [25]. Korea established GMP standards in 1977, which at that time were autonomous regulations. The Korean government in 1994 implemented mandated GMP production facilities for pharmaceutical manufacturers, and in 2008 introduced a new GMP system requiring validation. Later, in 2014, the Korean GMP system joined the Pharmaceutical Inspection Co-operation Scheme (PIC/S), and GMP in Korea was internationalized. It has been steadily revised in concert with WHO and global standards [26]. Some studies have examined changes to the GMP system by period and compared them between different countries [12, 25, 26]. As the second research question, this study determines whether there was a difference in growth according to the readiness of pharmaceutical companies for each change in regulations due to a change in Korean GMP policy.

### Price regulations and the introduction of new drugs

While the government is attempting to reduce the national healthcare burden by maintaining a policy to control the price of pharmaceuticals at an affordable level, pharmaceutical companies must raise prices to meet higher safety and effectiveness standards to compensate for high pharmaceutical R&D costs [6]. Previous studies have demonstrated that price regulation negatively affects the timing and occurrence of the launch of a new drug [11]. In addition, most drug price controls significantly impact the innovation strategies and financial status of pharmaceutical companies by reducing the revenue and R&D investment of companies through phenomena known as the cash-flow effect and the expected-profit effect [13, 27].

Korea’s National Health Insurance (NHI) system implemented a reimbursement reform through the DERP in 2006. The NHI was running a cumulative financial deficit due to high drug expenditures with a fast growth rate. The DERP aimed to reduce the health insurance budget. The main components of the DERP implemented in December 2006 were the introduction of a positive drug listing system, a requirement for submission of pharmacoeconomic evaluation data for new drug listings, and changes in the pricing policy for generic drugs. The requirement to submit economic evaluation data and negotiate with the regulatory authority complicated insurance registration of new drugs. Yang et al. [28] noted that the registration period was extended immediately after introducing the DERP. Son [29] reinvestigated the effects of new drug registration on licensing and insurance registration from 2007 to 2016 in Korea and found that the duration between regulatory approval and the reimbursement decision had decreased. Various stakeholders in the market adopt a new drug insurance listing, considering their strategic behaviour, and due to diverse factors have different listing periods [29]. As the third research question, this study seeks to determine how the new drug reimbursement registration period changed after implementing the DERP system.

## Methods

### Data

Patent application data from 1981 to 2016 were retrieved from the Korea Intellectual Property Rights Information Service (KIPRIS) to address research question 1. We selected the International Patent Classification (IPC) codes A61K (preparations for medical, dental, or toilet purposes) and C07 (organic chemistry) by the year of the filing. We excluded IPC codes A61K 6 (dental-related products) and A61K 7 (cosmetics), as well as the codes related to health foods.

For research question 2, we first obtained a list of the companies that pre-emptively prepared for the GMP changes from 1985 to 1990, before implementation of the mandatory GMP system, from the book, *The History of Korea Pharmaceutical Manufacturers Association (KPMA)’s 50 years* [30]. Sixteen foreign pharmaceutical factories and 34 domestic companies were recorded in this book. In this study, we included only domestic companies. Among the 34 domestic manufacturers, we excluded one company that had gone through a merger and two companies that do not currently produce pharmaceuticals. The total production of the remaining 31 companies accounted for 49.3% of all Korean pharmaceutical production, and 20 out of the 31 companies were in the top 30 pharmaceutical companies in Korea in 1994. Next, we assessed the production quantity of each pharmaceutical company from 1988 to 2017 for research question 2. We classified these 31 companies as group 1, the group that pre-emptively prepared for GMP regulations, while the remaining 230 companies were classified as group 2. Group 2 acquired GMP certificates only after the GMP regulations became mandatory in 1994. We then analysed the effects of three changes in GMP regulations (in 1994, 2008, and 2014).

We investigated the date of new drug approval by the Ministry of Food and Drug Safety and the start date of health insurance application coverage for research question 3 using the Health Insurance Review & Assessment Service (HIRA) database. A total of 780 new drugs were approved from 1989 to 2017, and we counted different ingredients on the active pharmaceutical ingredient (API) list for each drug as separate items. Among the 780 new drugs, those not covered by health insurance, such as over-the-counter (OTC) drugs and vaccines, were excluded from the analysis. We calculated the number of months between a product’s approval date and the insurance coverage application date. If the approval date was later than or the same as the commencement date of insurance benefits due to mergers and acquisitions or changes in import permits and manufacturing permits, we excluded that case. Finally, we selected 620 new drugs and calculated the period from new drug approval to insurance registration.

### Interrupted time series (ITS) analysis: methodology

ITS analysis is a quasi-experimental design that uses segmented regression modelling. Since ITS allows longitudinal data to evaluate intervention effects, it is an appropriate statistical method for observing changes after implementing an intervention, such as a government regulation [31]. ITS analysis can demonstrate an intervention effect by statistically measuring outcome variables at different time points before and after an intervention to compare the change in the level and trend of the outcomes [32]. In ITS, a time series is an iterative observation of a particular event collected at regular intervals divided into two or more segments at change points [33]. Two parameters, level and trend, identify each element of the time series. The level and trend indicate the series value at the beginning of a given time interval and the rate of change during a segment, respectively [33, 34].

Based on the literature, ITS analysis for a single intervention without a comparison group, called the single-intervention one-group model, can be explained as follows [31, 34, 35]: There are three variables for an ITS analysis in a single-intervention one-group model:

i. $T$: the time elapsed since the start of the study;
ii. $X_{t}$: a dummy variable representing the intervention (the pre-intervention period takes a value of 0, while the post-intervention period takes a value of 1);
iii. $Y_{t}$: the outcome at time *t*.

This ITS model has three measures of interest: the pre-intervention trend, the post-intervention trend, and the difference between the pre-intervention and post-intervention trends:

$$Y_{t} = \beta_{0} + \beta_{1} T + \beta_{2} X_{t} + \beta_{3} TX_{t} + e_{t} ,$$

where $\beta_{0}$ indicates the baseline level at $T = 0$, $\beta_{1}$ is the trend of the outcome variable until the beginning of the intervention, $\beta_{2}$ indicates the change in the level following the intervention, and $\beta_{3}$ represents the change in the trend following the intervention. In this model, $\beta_{1} + \beta_{3}$ represents the post-intervention trend, and $e_{t}$, the error term at time *t* indicates the random variability that the model does not explain.

In our ITS analysis, observed values are correlated with values at the immediately preceding point of time in time series data, such as the calendar data used in this study. We performed autocorrelation function (ACF) and partial ACF (PACF) analysis examining appropriate time lags to resolve this problem. Finally, for the analyses of regulations on patent applications, we applied two quarters of the time lag. For the analyses of GMP regulation, no time lag between regulation and results was applied. The final analysis was done using the maximum likelihood model to fit the data based on this time lag. We performed statistical analyses using SAS version 9.4 (SAS Institute, Cary, NC, USA) and R version 4.0.3 (R Foundation for Statistical Computing, Vienna, Austria) software.

## Results

### Research question 1: Did the introduction of the product patent system increase the number of patent applications filed by the pharmaceutical industry in Korea?

For ITS analysis of pharmaceutical patents, as the output variable, we counted the number of patent applications filed per quarter from 1981 to 1998. We used the following ITS analysis with a single-intervention one-group model to analyse the effects of implementing the product patent system, with July 1987 as the intervention time.

$$Y_{t} = \beta_{0} + \beta_{1} T + \beta_{2} X_{t} + \beta_{3} TX_{t} + e_{t}$$

*T*: the time elapsed since January 1981.

*X*_*t*_: a dummy variable indicating before (coded 0) and after (coded 1) enforcement of the product patent system in July 1987.

Count: number of patent applications per quarter.

$\beta_{0}$: the baseline level in January 1981.

$\beta_{1}$: the underlying trend before the introduction of the product patent system.

$\beta_{2}$: the level change after the introduction of the product patent system.

$\beta_{3}$: the slope change after the introduction of the product patent system.

$\beta_{1} + \beta_{3}$: the slope after the introduction of the product patent system.

**Fig. 1:**
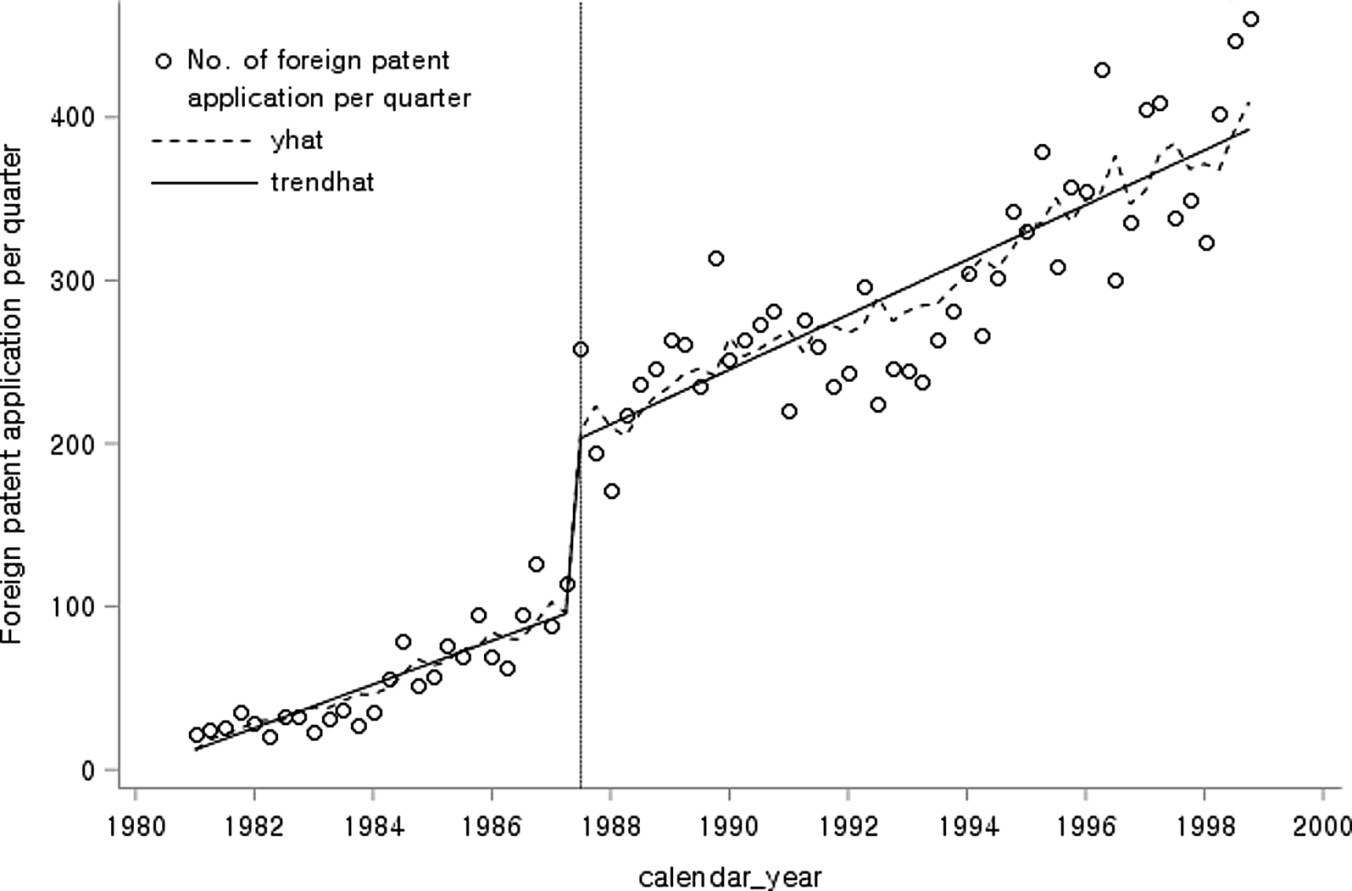
The graphical results of ITS analysis of the total number of patent applications

The results of ITS analysis show that $\beta_{0}$ = 12.2314, $\beta_{1}$ = 3.5109, $\beta_{2}$ = 95.6962, and $\beta_{3}$ = 3.9667. The *p*-values of $\beta_{1}$, $\beta_{2}$, and $\beta_{3}$ were 0.00, 0.01, and 0.01, respectively, all of which show less than the significance level of 0.05 (Fig. 1, Table 1). There was a level shift after the intervention (*p* < 0.05 for $\beta_{2}$), as well as a trend change after the intervention (*p* < 0.05 for $\beta_{3}$) (Table 1).

**Table 1:** The statistical results of ITS analysis of the total number of patent applications

Variable	Coefficient	SE	*t*	*p*
Intercept $\beta_{0}$	12.23	21.28	0.57	0.57
Baseline trend $\beta_{1}$	3.51	23.22	4.12	0.00
Level change after policy $\beta_{2}$	95.70	1.32	2.65	0.01
Trend change after policy $\beta_{3}$	3.97	1.52	2.61	0.01

SE: standard error

**Fig. 2:**
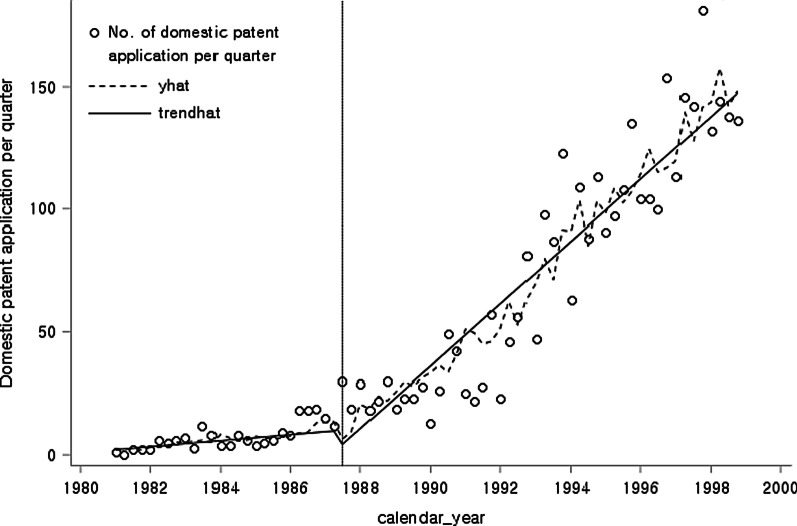
The graphical results of ITS analysis of the total number of Korean patent applications

To compare the effects of introducing the product patent system between Korean and foreign companies, we divide the total patent applications into Korean patent applications and foreign patent applications. The number of patents filed by Koreans increased after introducing the product patent system, with a change in slope (Fig. 2). ITS analysis indicated that $\beta_{0}$ = 1.7675, $\beta_{1}$ = 0.3087, $\beta_{2}$ = −8.6469, and $\beta_{3}$ = 2.8654. The *p*-values for $\beta_{1}$ and $\beta_{2}$ were 0.3693 and 0.5732, respectively, which were greater than 0.05, and thus were not statistically significant (Table 2). However, the regression coefficient $\beta_{3}$ was significant, with a *p*-value of less than 0.0001, which means that the changes to the patent law system significantly affected the trend in Korean patent applications.

**Table 2:** The statistical results of ITS analysis of the total number of Korean patent applications

Variable	Coefficient	SE	*t*	*p*
Intercept $\beta_{0}$	1.7675	8.7580	0.02	0.8407
Baseline trend $\beta_{1}$	0.3087	9.5650	−0.90	0.3693
Level change after policy $\beta_{2}$	−8.6469	0.5452	0.57	0.5732
Trend change after policy $\beta_{3}$	2.8654	0.6265	4.57	< 0.0001

SE: standard error

**Fig. 3:**
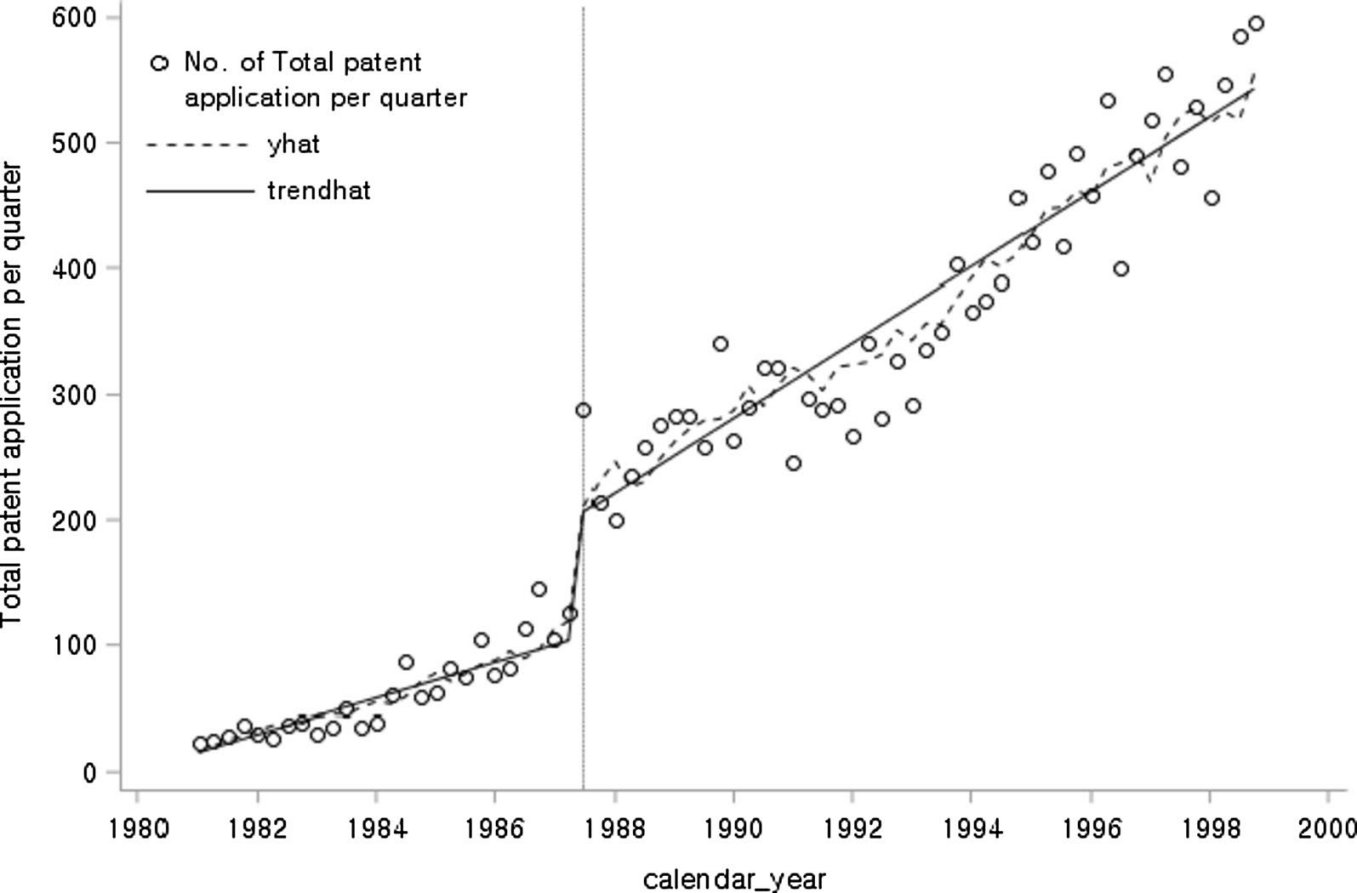
The graphical results of ITS analysis of the total number of foreign patent applications

Changes in the number of foreign patent applications resulted in changes in the level after the intervention (Fig. 3). Table 3 shows that $\beta_{0}$ = 8.9220,${ }\beta_{1}$ = 3.3391, $\beta_{2}$ = 103.3646, and $\beta_{3}$ = 0.8637. The regression coefficients of $\beta_{1}$ and $\beta_{2}$ were statistically significant, with *p*-values of less than 0.05. The *p*-value for $\beta_{3}$, was 0.4914, which is greater than the significance level of 0.05, so $\beta_{3}$ was not significant (Table 3). A rapid increase in the level of foreign patents followed the introduction of the product patent system, but the trend was not statistically significant.

**Table 3:** The statistical results of ITS analysis of the total number of foreign patent applications

Variable	Coefficient	SE	*t*	*p*
Intercept $\beta_{0}$	8.9220	17.5990	0.51	0.6139
Baseline trend $\beta_{1}$	3.3391	20.4569	5.05	< 0.0001
Level change after policy $\beta_{2}$	103.3646	1.1236	2.97	0.0041
Trend change after policy $\beta_{3}$	0.8637	1.2481	0.69	0.4914

SE: standard error

The three above-mentioned ITS analyses show that the introduction of the product patent system in 1987 led to significant increases in the level change and the trend change of the total patent applications, and a more positive effect in the trend change of Korean patent applications.

### Research question 2: Did companies that pre-emptively invested in GMP facilities before mandatory GMP maintain sustainable growth in Korea?

There were several changes to the GMP policy in Korea: mandatory implementation of GMP in 1994, pre-approval GMP evaluation for manufacturing items in 2008, harmonization with the PIC/S GMP guidelines in 2014, and periodic GMP evaluation carried out on the dosage forms of all manufacturing sites.

Before GMP was legally mandated, 31 companies pre-emptively invested and were GMP-certified during the period from 1985 to 1990. These pre-emptively prepared companies (group 1) had a total production value of 138 trillion won over 30 years and an average annual growth rate of 6.9% over 30 years. However, the non-pre-emptively prepared companies (group 2) had a total production value of 135 trillion won and a growth rate of 8.4% (Table 4). In terms of production performance, companies that pre-emptively invested before mandatory GMP implementation in 1994 were predominant in the initial market. In 2008, when capital investment was required due to the mandatory GMP validation, the 31 pre-emptively prepared companies increased their output further. In 2014, the PIC/S GMP system did not require capital investment, so the impact on the system was negligible. Instead, it was estimated to have a reverse effect due to the price slashing of finished drugs in 2012.

**Table 4:** Total production of group 1 and group 2 companies

	Production amount (Bill. )						
	1988–1993	1994–1998	1999–2002	2003–2007	2008–2012	2013–2017	Total
Group 1 (CAGR)	10 809 (13.25%)	15 582 (6.45%)	14 697 (4.70%)	24 561 (9.98%)	35 537 (0.59%)	36 837 (4.37%)	138 022 (6.90%)
Group 2 (CAGR)	9500 (16.98%)	15 358 (4.44%)	13 878 (4.30%)	24 338 (8.11%)	31 795 (2.66%)	40 034 (5.03%)	134 904 (8.40%)
Difference (CAGR)	1309 (−13.78%)	224 (−1.50%)	819 (−5.90%)	223 (−0.90%)	3742 (−11.80%)	(−)3197 (7.98%)	3118 (−2.30%)

CAGR: compound annual growth rate

**Fig. 4:**
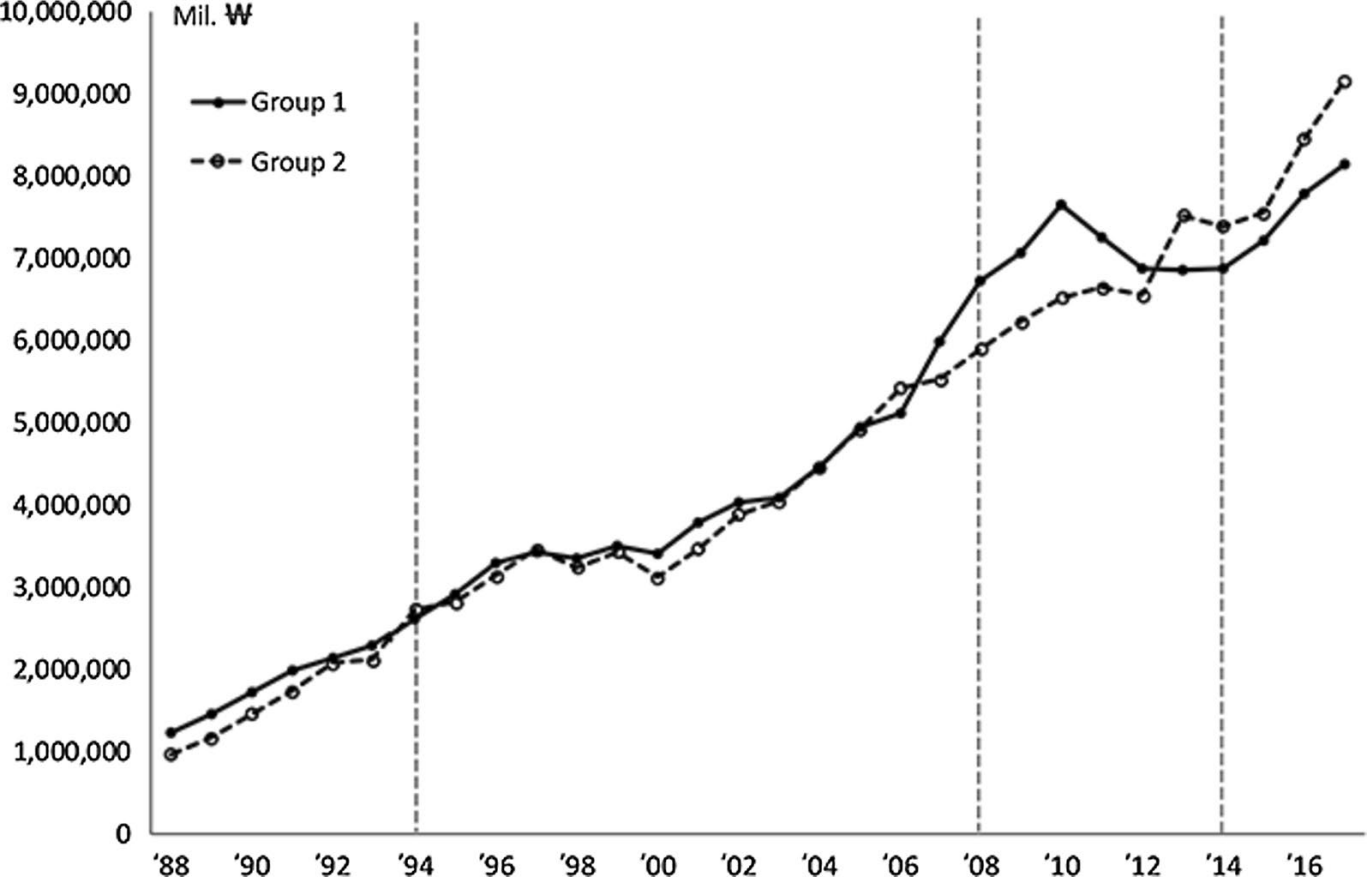
Total production by group 1 and group 2 companies over time

Figure 4 shows the changes in production after the three most significant changes in GMP regulations.

We used ITS analysis again to see whether pre-emptive investments in GMP facilities affected the sustainable growth of Korean pharmaceutical companies. Since this ITS analysis has three interventions and two groups, group 1 with pre-emptive investments in GMP facilities and group 2 without pre-emptive investments, we need to extend the single-intervention one-group model of Section 2 to a model with multiple interventions and a comparison group [34, 35].

In this study, we investigate the effects of three sequential GMP policies: policy 1 represents the mandatory GMP policy in 1994, policy 2 represents the requirement for pre-approval for GMP evaluation of manufacturing items in 2008, and policy 3 represents PIC/S GMP in 2014. The final ITS model with three interventions and a comparison group was as follows:

$$Y_{t} = \beta_{0} + \beta_{1} T + \beta_{2} X_{1t} + \beta_{3} T_{1t} X_{1t} + \beta_{4} X_{2t} + \beta_{6} X_{3t} + \beta_{7} T_{3t} X_{3t} + \beta_{8} Z + \beta_{9} ZT + \beta_{10} ZX_{1t} + \beta_{11} ZT_{1t} X_{1t} + \beta_{12} ZX_{2t} + \beta_{13} ZT_{2t} X_{2t} + \beta_{14} ZX_{3t} + \beta_{15} ZT_{3t} X_{3t} + e_{t} ,$$

where $X_{1t}$, $T_{1t} X_{1t}$, $ZX_{1t}$, and $ZT_{1t} X_{1t}$ represent the policy 1 period; $X_{2t}$, $T_{2t} X_{2t}$, $ZX_{2t}$, and $ZT_{2t} X_{2t}$ represent the policy 2 period; and $X_{3t}$, $T_{3t} X_{3t}$, $ZX_{3t}$, and $ZT_{3t} X_{3t}$ reflect the policy 3 period. $Z$ is a dummy variable denoting the cohort assignment (pre-emptive investments in GMP facilities or not), and $ZT$, $ZX_{1t}$, $ZT_{1t} X_{1t}$, $ZX_{2t}$, $ZT_{2t} X_{2t}$, $ZX_{3t}$, and $ZT_{3t} X_{3t}$ are all interaction terms between previously described variables. The coefficients $\beta_{0}$ to $\beta_{7}$ represent the levels or trends of the control group (non-pre-emptive investments in GMP facilities), and the coefficients $\beta_{8}$ to $\beta_{15}$ represent the levels or trends of the treatment group (pre-emptive investments in GMP facilities).

**Fig. 5:**
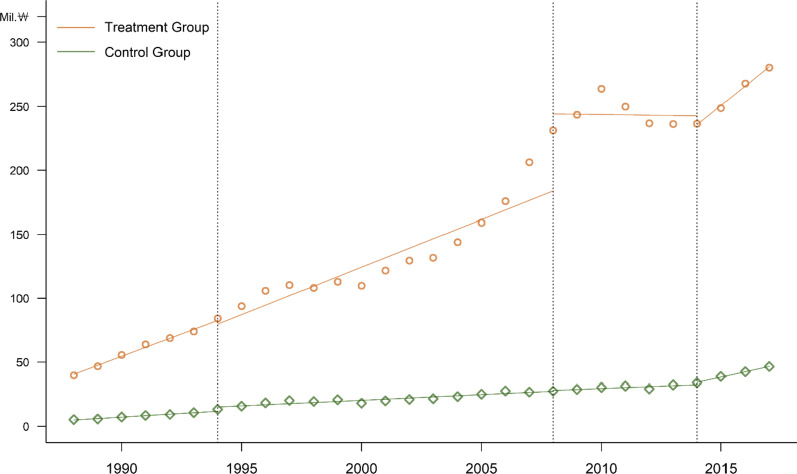
The effects of the GMP policy on the production of group 1 and group 2 by ITS analysis

In this ITS model, there are 30 full measures of interest: the pre-intervention, policy 1, policy 2, and policy 3 trends for the treatment group and the control group; the differences between groups in their trends in each of these periods, the differences between each period’s trends for the treatment group and control group (pre-intervention versus policy 1, pre-intervention versus policy 2, pre-intervention versus policy 3, policy 1 versus policy 2, policy 1 versus policy 3, and policy 2 versus policy 3); and the contrast between groups for each of these periodic comparisons. The regression output provides these eight measures: $\beta_{1}$, $\beta_{3}$, $\beta_{5}$, $\beta_{7}$, $\beta_{9}$, $\beta_{11}$, $\beta_{13}$, and $\beta_{15}$. The remaining 22 composite measures of interest can be calculated using those eight measures. Figure 5 and Table 5 show the results of the GMP policy on the production of group 1 and group 2 by the ITS analysis.

**Table 5:** The effects of the GMP policy on the production of group 1 and group 2 by ITS analysis

Variable		Coefficient	SE	*t*	*p*
Control group	$\beta_{0}$	3 921 915	7 296 197	0.54	0.5936
	$\beta_{1}$	1 121 879	1 873 490	0.6	0.5524
	$\beta_{2}$	3 613 799	7 193 712	0.5	0.6179
	$\beta_{3}$	−241 415	1 944 213	−0.12	0.9017
	$\beta_{4}$	769 559	8 308 389	0.09	0.9266
	$\beta_{5}$	−148 447	1 944 213	−0.08	0.9395
	$\beta_{6}$	−1 645 401	11 149 497	−0.15	0.8834
	$\beta_{7}$	3 412 147	3 974 273	0.86	0.3952
Treatment group	$\beta_{8}$	29 594 918	10 318 380	2.87	0.0063^*^
	$\beta_{9}$	5 905 425	2 649 515	2.23	0.031^*^
	$\beta_{10}$	−6 906 227	10 173 446	−0.68	0.5008
	$\beta_{11}$	644 845	2 749 532	0.23	0.8157
	$\beta_{12}$	67 319 195	11 749 837	5.73	< 0.0001^*^
	$\beta_{13}$	−7 534 099	2 749 532	−2.74	0.0088^*^
	$\beta_{14}$	−20 685 531	15 767 771	−1.31	0.1964
	$\beta_{15}$	11 963 845	5 620 471	2.13	0.0389^*^

*p < 0.05 (two-tailed test)

Group 1, which pre-emptively prepared for GMP, had a beginning average production level that was significantly greater than that of group 2, which did not prepare in advance (*p* = 0.0063 for $\beta_{8}$). Before policy 1, the GMP mandate implemented in 1994, the level and slope of the treatment group were significantly different (*p* = 0.0063 for $\beta_{8}$, *p* = 0.031 for $\beta_{9}$) from those of the control group. The average production level increased significantly immediately after the implementation of policy 2, which in 2008 was expanded to include pre-approved GMP evaluation of manufacturing items (*p* < 0.0001 for $\beta_{12}$).

After the implementation of policy 2, there was a significant difference (*p* = 0.0088 for $\beta_{13}$) between the treatment group and the control group in the slope change (Table 5). Even after the implementation of policy 3 (2014 PIC/S GMP), the difference in slope between the treatment group and the control group from the previous period was significant (*p* = 0.0389 for $\beta_{15}$).

In conclusion, companies that responded to the GMP system in advance showed excellent pharmaceutical production performance. The growth trend of the treatment group also improved more rapidly after the implementation of Policies 2 and 3. After implementing policy 1, the growth trend did not change significantly because, before policy 1 implementation, the growth trend in the treatment group was sufficiently large. Nevertheless, there was a significant difference in the temporary growth level immediately after implementation of policy 2 because the production of three batches before drug approval was done for compulsory validation.

### Research question 3: Did the DERP delay the introduction of new drugs in Korea?

The DERP, which is considered one of the most significant changes to the domestic insurance drug pricing system over the past 30 years, was amended on 29 December 2006. This study investigated the impact of the DERP on new drug development using ITS analysis of the change in the starting date of insurance coverage of the new drug. The intervention time for the ITS analysis of the DERP was set to January 2007 because the regulatory implementation date was the end of December 2006. According to the health insurance application date, a total of 321 new drugs were included in this analysis for the period before 2007, and 297 from January 2007 until December 2017. In addition, the average duration of insurance coverage of the 618 items in the entire period was 19.9 months; from 1989 to 2006, it was 18.0 months; and after 2007, 22.0 months.

**Fig. 6:**
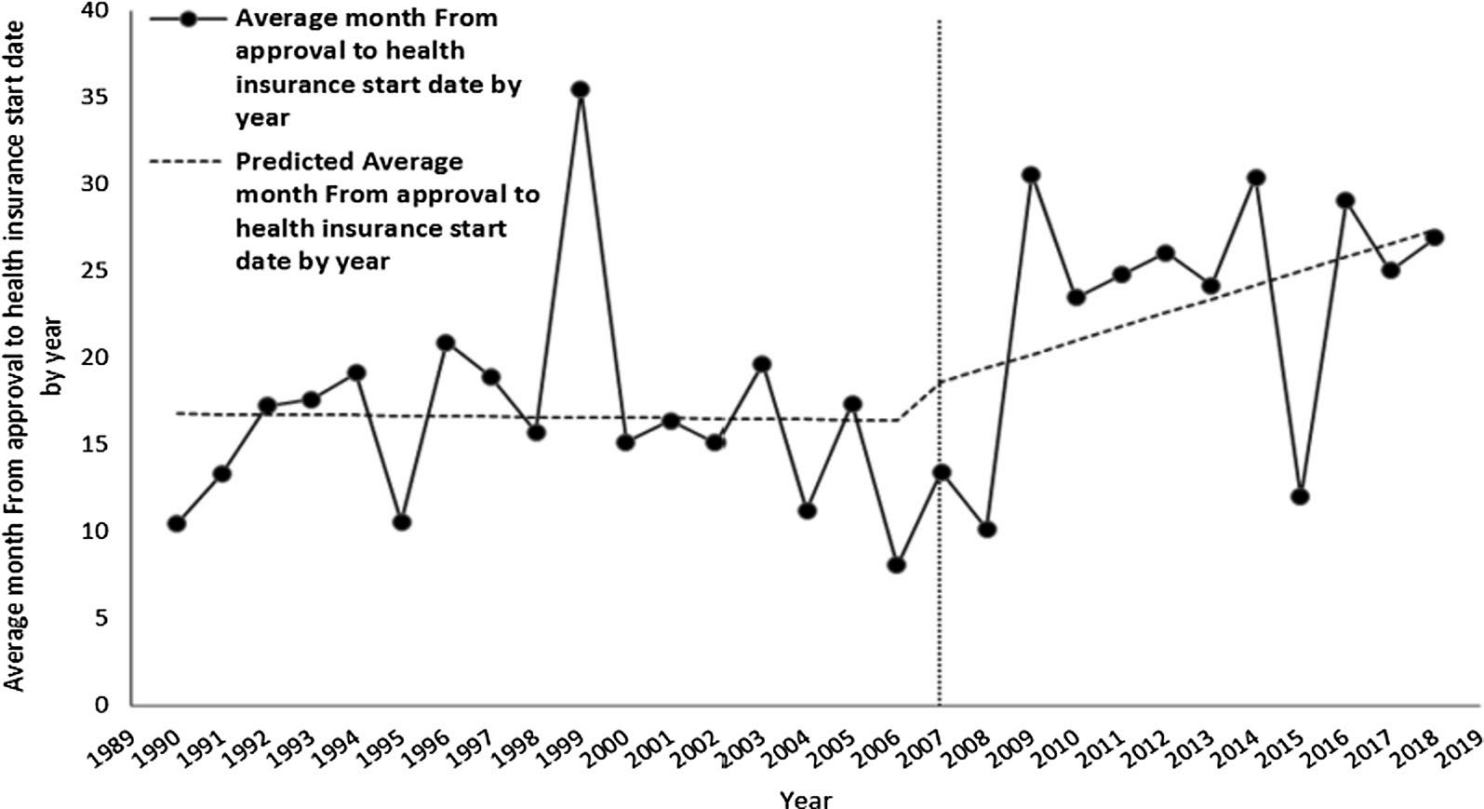
Average periods from drug approval to health insurance start date

After implementation of the DERP system, health insurance coverage for new drugs was delayed by about 4 months. The average periods from approval of the health insurance start day were analysed using the final ITS model, as shown in Fig. 6. The results of ITS analysis showed that $\beta_{0}$ = 16.8413, $\beta_{1}$ = −0.0242, $\beta_{2}$ = 1.3867, and $\beta_{3}$ = 0.8236, as shown in Table 6, and none of the *p*-values for $\beta_{1}$, $\beta_{2}$, or $\beta_{3}$ were statistically significant. Note that although 22.0 months, the average registration time after DERP implementation, is longer than the 18.0-month average registration time before the DERP policy, the ITS analysis showed no statistical evidence that DERP had resulted in a delay in bringing new drugs to market. It is likely that the several outlier years, such as 1999, 2009, and 2015, make the variance too large for the statistical test to be significant.

**Table 6:** Average number of months from drug approval to health insurance start date

Variable	Coefficient	SE	*p*-value
Intercept $\beta_{0}$	16.8413	3.0568	< 0.0001
Baseline trend $\beta_{1}$	−0.0242	0.2983	0.9354
Level change after policy $\beta_{2}$	1.3867	4.6459	0.7653
Trend change after policy $\beta_{3}$	0.8236	0.5856	0.1596

SE: standard error

Although there was a tendency towards an increase in the time between the date of approval of a new drug and the starting date of health insurance coverage, the variation in the average amount of elapsed time was considerable, so the results were not statistically significant. Therefore, the results differed from our expectations. However, when we analysed the data in detail, we made the following discoveries. First, the listing period is shortened if a pharmaceutical company accepts a lower price than the expected price of a new drug through negotiation with the regulatory authority. Therefore, the listing period varies depending on the drug company's strategy and the product's cost structure. Second, we also consider that the linkage system between drug approval and price evaluation, implemented in 2014 to improve new drug accessibility and supply new drugs to patients quickly, may be one reason for the lack of statistical significance of the ITS analysis. According to the HIRA, immediately after implementation of the linkage system between drug approval and price evaluation, the drug approval and reimbursement registration period for new drugs was shortened to about 100 days [36].

## Discussion

Since the introduction of the product patent system, the number of Korean patent applications filed by domestic companies has increased significantly. When the product patent system was introduced in 1987, many people were worried that the intense competitiveness in patent rights of foreign companies would allow them to dominate domestic companies [30]. However, this study shows that domestic companies achieved high growth by developing new drugs, evidenced by the expanding number of patent applications. This finding implies that the product patent policy has reinforced the innovative capabilities of domestic pharmaceutical companies in Korea.

Given the mandatory GMP system changes, pharmaceutical companies need to invest heavily in GMP facilities, and only financially sound companies were able to invest in response to the change. However, this study showed that the growth of pre-emptively invested companies in Korea was considerable. This result implies that pharmaceutical companies should invest in preparation for institutional changes, such as GMP.

Unlike previous studies, which claimed that the price regulation system delayed the registration period for new drugs [29], this study found no significant delay in new drug registration during the 10-year period after implementation of the DERP system in Korea. The results indicated that Korea compensated for the possible delay of new drugs due to DERP through other policies, such as the linkage system between drug approval and price evaluation, and through negotiations between the companies and the regulatory authorities [29]. Pharmaceutical companies are sensitive to delays in the launch of new drugs, since the patent and marketing efforts for the new drug continue regardless of whether or not the product is on the market. In addition, if the drug is more cost-effective than other available options on the current market, a delay in launching new drugs may be costly to consumers [11]. This study suggests that national regulatory authorities should supplement drug pricing policies to help pharmaceutical companies with this issue.

This study has potential limitations. First, other confounding policy factors over the long term may have influenced our analysis results. Internal factors, including R&D intensity, company size, human resources, and company strategy, could also affect the long-term trend in Korean pharmaceutical companies. Therefore, analysis of the confounding policy factors and the internal factors via general morphological analysis (GMA) and regression analysis could provide more information about the changing pattern of the companies and a more precise relationship between confounding factors. Second, our analysis was retrospective in nature, so it was limited in not predicting the future effects of the policy prospectively. However, the retrospective insight could reflect future national-level policy establishment implications by extrapolating the past long-term policy outcome. Third, this study mainly considers the impacts of the major regulations from a pharmaceutical industry perspective. Considering that public benefits are one of the main reasons for policy establishment, future research needs to analyse the changes affecting public consumers after policy implementation, thus providing balanced insight from both the industry and customer perspectives. Finally, although nine or more observations in both pre-intervention and post-intervention are encouraged for ITS analysis [37], two post-intervention periods in our analysis on GMP policy involved fewer than nine observation points. Thus, it may have limited statistical power.

## Conclusion

This study compared the outcomes before and after implementation of major regulations and assessed long-term changes by looking at data for an 18-year period encompassing the introduction of the product patent system, a 30-year period during which the GMP system was implemented, and 30 years that included changes to the pricing system. We investigated the effects of these three crucial policies from a long-term perspective using a single methodology, ITS analysis. To the best of our knowledge, this study is the first long-term study of the impact of significant regulations on the Korean pharmaceutical industry.

The average yearly growth rate in pharmaceutical products from 1988 to 2017 was 7.59%, which is greater than the 5.26% GDP growth during those years in Korea, one of the world’s fastest GDP growth countries in that period [38]. This study showed that Korean pharmaceutical companies actively responded to necessary regulatory policies that guided and regulated the significant growth of the Korean pharmaceutical industry over the past 30 years.

The Korean government has tightened regulations for the public interest, securing drug safety, and reducing budgets. Korean pharmaceutical companies have established strategies to respond to such policies, which have led to pharmaceutical innovation over a 30-year period. As a result, Korean pharmaceutical companies improved the quality of pharmaceuticals and developed excellent new drugs in terms of pharmacoeconomics and the national economy. This study implies that the fast growth of the pharmaceutical industry in Korea was possible because the regulatory authorities protected consumer health, alleviated cost burdens, promoted pharmaceutical innovation, and improved the global competitiveness of domestic pharmaceutical companies.

## Data Availability

The datasets generated and analysed in this study are available from the authors on reasonable request.

## Abbreviations

API: Active pharmaceutical ingredient
DERP: Drug Expenditure Rationalization Plan
GMP: Good Manufacturing Practice
HIRA: Health Insurance Review & Assessment Service
IPC: International Patent Classification
ITS: Interrupted time series
KIPRIS: Korea Intellectual Property Rights Information Service
KPMA: Korea Pharmaceutical Manufacturers Association
NHI: National Health Insurance
OTC: Over-the-counter
PCT: Patent Cooperation Treaty
PIC/S: Pharmaceutical Inspection Co-operation Scheme
